# How Consumer Behavior in Daily Food Provisioning Affects Food Waste at Household Level in The Netherlands

**DOI:** 10.3390/foods8100428

**Published:** 2019-09-20

**Authors:** Kim Janssens, Wim Lambrechts, Annet van Osch, Janjaap Semeijn

**Affiliations:** Open University of the Netherlands, Valkenburgerweg 177, 6401 DL Heerlen, The Netherlands; wim.lambrechts@ou.nl (W.L.); janjaap.semeijn@ou.nl (J.S.)

**Keywords:** food waste, food waste behavior, consumer behavior, household food waste prevention, sustainable grocery management, grocery retail

## Abstract

Food production and consumption have remarkable negative environmental effects, in particular food waste. Food waste occurs throughout the entire food system, but households make the largest contribution. Reducing unnecessary waste of food represents a crucial step toward overcoming global issues of food waste, hunger, and climate change. Identifying barriers in reducing food waste is important not only to government and policymakers, but also to food producers, retailers, and marketers. Therefore, the objective of this research was to find out how consumer behavior in daily food provisioning affects food waste. An online survey was set up to question Dutch consumers (partly) in charge of the household’s food management. A total of 211 consumers participated in answering questions on household composition, food management behavior (e.g., food purchase planning) and food waste awareness (i.e., concern about wasting food and intention not to waste food). Results show that purchase behavior in-store was the main driver of food waste. Specifically, participants indicated that buying more food than needed often had led to food waste. In addition, intention not to waste food acted as a moderator in the relationship between planning behavior and food waste. Age appears to have a diminishing impact on wasting food.

## 1. Introduction

Food production and consumption, and more particularly food waste, are responsible for striking negative effects on the environment [1]. A recent EU-project [2] stated that 89 million tons of food are wasted each year and that the total amount of food waste for 2020 could rise by an additional 40%. Food losses and food waste occur throughout the entire food supply chain (FSC): households account for 53%, manufacturers for 30% (production and processing), retailers for 5%, and food service for 12% [3]. Based on Searchinger et al. [4] Europe is responsible for 22% of the global food waste (with 11% during the consumption stage). There is a consensus in available literature that households contribute greatly to the total amount of food waste, specifically in the Netherlands [2,5,6,7]. Food waste produced by Dutch households was 576 kg per capita in 2006 (while the EU average was 423 kg) [5]. In September 2015 several studies addressing this topic led to Sustainable Development Goals (SDGs) to which the EU committed. One of the main goals is to halve the food waste per capita at retail and consumer level by 2030 and to reduce overall food losses in the food supply chain [8].

Given the high amount of food waste at household level, prevention of food waste at the final stages of the food supply chain is of greatest importance to limit negative effects on the environment [9]. When households waste food all (fossil) energy and greenhouse gas emissions put into its production and distribution serve no purpose [10,11]. Apparently, the majority of Europeans point to individual responsibility when it comes to ways of reducing food waste, with 63% saying that better food-related practices in terms of planning and shopping would help to reduce waste [12]. However, in spite of this concern, the level of food waste continues to be very high.

Recently, two systematic reviews [2,13] have highlighted the importance of having a better understanding of behaviors contributing to household food waste. This knowledge should increase theoretical insights and assist to develop practical implications. These findings can support organizations, especially retailers, in developing more effective measures countering food waste at household level [14]. Besides, specifying food waste behavior(s) can also help develope counter food waste measurements [15].

The objective of this research, therefore, is to find out how consumer behavior in daily food provisioning affects food waste. From a theoretical perspective, this study extends recent research [14,16,17] explaining food waste behaviors combining classic psychosocial factors [18] with the role of household food-related practices. These studies identified different relationships between behavior and food waste, however, with contradicting findings. For instance, Stancu et al. [16] found that food purchase planning behavior only made an indirect contribution to the amount of food waste, whereas Stefan et al. [17] showed that planning routines directly contribute to lowering food waste at home. Additionally, Romani et al. [14] view consumption of leftovers as the least important factor in countering food waste. Contrary, Stancu et al. [16] found that left-over consumption behavior describes one third of the variance in reported food waste.

In addition to the scientific contribution, the results of this study are relevant to policymakers, suppliers, and retailers. A good understanding of Dutch household behavior influencing the planning, purchase, storage, and preparation of food can contribute to essential knowledge necessary to ensure that initiatives such as interventions, development of products, and campaigns will succeed. Furthermore, this study serves the public interest by contributing to knowledge on how to reduce food waste in general.

## 2. Theoretical Background

### 2.1. Food Waste and the Environment

Food production, consumption, and the waste of food are responsible for negative effects on the environment [1]. The primary production of food requires the use of resources such as fuel, land, water, and raw materials. Food losses and wastes are accompanied by various environmental impacts, such as soil erosion, deforestation, water, and air pollution. Besides, greenhouse gas emissions occur during the different upstream and downstream stages in the FSC, namely pre-production, production, post-production, consumption, loss, and waste of food [18,19,20]. When food is wasted instead of consumed, the environmental impact of food production and consumption is even bigger because of the processing of the waste [10]. In addition, food waste is also water waste, because of the large amounts of water that is used during the producing of food [7]. Given the high amounts of food waste in the final stages of the FSC, the prevention of food waste at these stages is of great importance to prevent further climate change [9].

### 2.2. Prevention of Food Waste

The drive to target food waste stems from increasing concerns about resource conservation, food security, and the environmental and economical costs of food waste [21]. Hence, prevention of food waste is found to be one of the most promising means to achieve environmental impact savings [16]. The global population will only increase, which implies that more people have to share the available food. The reduction of food waste is seen as a strategy in order to feed the increasing global population. In addition, there is a cost advantage for consumers, as purchased but not eaten food is a waste of money [16]. The negative impacts of food waste, but also the advantages of wasting less food, call for more attention toward ways to reduce food waste in general. Opportunities to reduce food waste include changing consumer perceptions about food and food waste [22], reducing overstock, reducing portion sizes in restaurants [20], utilizing packaging and processing technologies that help keep food fresh for longer [23], and clarifying the meaning of sell-by and use-by dates for consumers [24]. Opportunities to reduce food waste include complex customer behaviors such as planning, purchase, storage, and cooking behaviors [23].

### 2.3. Food Waste at Household Level

Although consumer food waste has increasingly received attention, its complex nature is far from unraveled [25,26]. Kosseva et al. [7] indicated that reducing food waste in developed countries is a big challenge because it is related to the behavior and attitudes of consumers. Still, little is known about the underlying factors that can explain food waste behaviors and practices. The literature seems to lack a clear understanding of the reasons of household food waste. Moreover, there are only a few studies with a focus on food waste and its relation to consumer behaviors [17]. It is possible to distinguish food-related behavioral factors directly affecting food waste from a wide range of other factors [25]. These factors can be personal (such as being poor or rich) or product specific (such as large packages) [14]. Waste and Resources Action Programme (WRAP) [27] developed a conceptual framework that highlights the different factors influencing food waste. There are several reasons why food is wasted, and multiple behaviors lead to the waste of food. In this study, food waste related behaviors involve the planning, shopping, storage, preparation, and consumption of food.

### 2.4. Food Management Behaviors

Consumer behavior is considered the main cause of food waste in developed countries [22]. Bravi et al. [28] identify three main behavioral antecedents for food waste among a sample of young Italian consumers: over preparation, excessive purchase and inappropriate conservation. Avoiding food waste is a responsibility of the consumer (e.g., regarding conserving food in an appropriate manner), however, regarding purchasing behavior, retailers also play an important role (e.g., avoiding excessive purchasing). With regard to producer and retailer responsibility in food waste, particular attention is needed towards production processes, portions and packaging of food, as well as discouraging excessive purchasing. Large quantities of food products available in-store and a wide range of food products offered lead to higher food waste. More replenished supplies increase the likelihood of some of those products reaching the sell-by date before being sold and wasted [29]. A recent exploratory study in the Dutch context for example, pointed out that smaller amounts of food were wasted at the household level, when consumers used frozen food equivalents instead of fresh or ambient food equivalents. This could be an additional lever to encourage consumers to avoid food waste [30].

Often, the theory of planned behavior (TBP) [31] is integrated in available research on consumer perceptions and behaviors regarding food waste [32]. TPB explains that behavioral intention (i.e., the willingness to behave in a certain way) is the primary cause of behavior (i.e., the action taken) [31]. TPB states that behavior is best explained through the intention a person has to actually show that behavior. As consumers are generally waste aversive [33] there is reason to believe that intentional processes may drive their food waste behavior. Consumers, in fact, perceive food waste as a food-related behavior more than as an environmental or a social behavior [21,27,34,35] and are not yet (fully) aware of the environmental or social impacts. Food waste can be seen as the last stage of decision-making in the food process [36] and waste behaviors have a strong connection with other food-related behaviors. All of the food-related behaviors therefore may be important in explaining food waste and will be further explained in the next section.

Food (management) behaviors relate to many different aspects of food product journeys: planning, shopping, storage, preparation, and consumption of food. Food waste is an outcome of the way households deal with these different stages. For instance, not making a shopping list before shopping may result in buying food that is already in the pantry or fridge during the shopping stage, which subsequently may result in failure to consume food before its expiry date. Alternatively, poor planning may imply that the consumption of food present in the household is not adequately stored and as a consequence this food runs past its expiry date. In both cases, food waste is associated with storing food for too long [25]. In conclusion, by the time food is thrown away, the opportunity to prevent the waste has usually passed [27]. The most commonly cited consumer food management behaviors can be categorized into planning, (in-store) purchase, storage, preparation, serving, and leftover consumption practices [25,36]. Serving behaviors will not be discussed in this study. Stefan et al. [17] conclude that planning and shopping routines explain most of the variance in food waste. At the purchase stage, consumers often rely on food shopping routines and admit to regularly buying more food than needed [37] or buying food products they never use, thereby increasing possible food waste. By contrast, planning routines such as checking the inventory level, making shopping lists, or planning meals in advance help consumers to limit food waste.

We expect and hypothesize that planning routines (e.g., checking inventory, making shopping lists, planning meals ahead) will have a negative influence on the amount of food wasted (i.e., lowering the amount of food waste), while certain shopping routines (e.g., buying too much food or unintended products) should have the opposite effect:

**Hypothesis** **1** **(H1).** *Food storage behavior (FSB) negatively influences the (reported) amount of Food Waste (FW).*

**Hypothesis** **2** **(H2).** *Food purchase planning behavior (FPB) negatively influences the (reported) amount of FW.*

**Hypothesis** **3** **(H3).** *Food purchase behavior in-store (FPBI-S) positively influences the (reported) amount of FW.*

**Hypothesis** **4** **(H4).** *Food planning preparation behavior (FPPB) negatively influences the (reported) amount of FW.*

**Hypothesis** **5** **(H5).** *Leftover consumption behavior (LCB) negatively influences the (reported) amount of FW.*

### 2.5. Intention Not to Waste Food

Several studies argue that food decisions are influenced by deep-rooted judgments such as emotions, hunger, values, and habits [34,38]. This leads to a high uncertainty level to characterize consumers’ food choices [39]. These behavioral aspects indicate that the performed behavior can generate a lot of outcomes. Consumers face a set of personal motivations that can(not) be in line with the intention to prevent or reduce food waste. For that reason, food waste-related motivation can cause an intention-behavior gap [40]. The intention-behavior gap is the more general finding that people’s motivations do not accord with their behavior [41]. Furthermore, Setti et al. [39] argue that a gap between food choices and expected consequence (food waste) is a behavior-outcome gap and can further influence consumers’ decision-making. Graham-Rowe et al. [40] conclude that the strength of the intention-behavior relationship is likely to be moderated by whether or not the person actually had control over the behavior. This problem can emerge when other members of the family show behaviors that are not in line with the behaviors of the respondent. Summarizing, several studies argue that an intention to avoid or reduce food waste is significantly related to less food waste [17,40]. According to these studies, higher intention not to waste leads to lower amounts of food waste. Therefore, the following hypotheses are included to find out whether results in the Netherlands correspond with previous research in other countries:

**Hypothesis** **6** **(H6).** *Respondents that intend to not waste food (INW), report lower amounts of FW.*

**Hypothesis** **6a** **(H6a).** *Intention not to waste food (INW) has a positive moderating impact on FSB, FPB, FPBI-S, FPPB, and LCB and the (reported) amount of FW.*

### 2.6. Concern about Food Waste

Concern about food waste may be related to personal values and may influence attitudes and behavior, such as food waste behavior. Grunert et al. [42] examined consumers’ underlying motivations and highlighted the influence of personal values embedded in these motivations. However, there is still a lack of literature examining environmental/food waste concern and to what extent waste concern may lead to adopting behaviors in order not to waste food [43]. Evans [37] showed that some people may experience a conflict in their attitudes regarding food waste. On the one hand, consumers seem to hold negative personal attitudes and personal norms regarding throwing food away. On the other hand, they may not want to risk their health by eating leftovers or foods that have passed their use-by dates. The latter concern, however, appeared less strongly related to food waste behavior than the former [44]. Some studies state that environmental concerns are not related to the amount of reported food waste. For example, Quested et al. (WRAP) [27] argue that the link between food waste and environmental impact is not firmly established in people’s minds. However, a few studies show that the environmental concern of individuals can be an important indicator impacting food waste behavior [45]. In fact, recent studies showed that a greater awareness concerning food waste can be positively linked to a different purchase behavior [46]. Taking the environmental concerns into consideration on reported food waste and food waste prevention behaviors, this study aims to clarify whether concern about food waste influences the amount of food wasted:

**Hypothesis** **7** **(H7).** *Respondents that are concerned about food waste have more intentions not to waste food.*

**Hypothesis** **7a** **(H7a).** *Respondents that are concerned about food waste report lower amounts of food waste.*

### 2.7. Socio-Demographics

Socio-demographic factors may be associated with food waste behavior. Koivupuro et al. [47] combined a diary method with a questionnaire to analyze food waste in the Finnish household context. They found out that the factors correlating to food waste the most are household size, gender of the person responsible for groceries, frequency of buying discounted products, as well as the respondent’s views on possibilities to reduce waste and the purchasing behavior (i.e., buying particular food packet sizes). A characteristic identified in previous studies is household size: the larger the household, the more food is wasted [44,48]. Members of larger households are, however, responsible for less waste per capita than members of smaller households [27]. Besides, Parizeau et al. [48] argue that households with more children produce more food waste. Parents reported difficulties in predicting how much food children would eat or who would be eating at home [37]. These findings are not in line with findings from the Organisation for Economic Co-operation and Development (OECD) [49] claiming that couples in the Netherlands with children waste 25.7% and couples without children waste 30.6%. The reported amounts in the Netherlands are contradictive to other countries, where couples without children waste less food. The age of the person responsible for food preparation seems to be related to the amount of food waste; the older, the less food is wasted [27]. Older people’s experiences with food shortage situations, such as during World War II, may explain this relationship.

A number of studies suggest that women waste more than men, whereas other studies state that females are more likely to reduce waste than males [46,50]. There is no consensus to what extent gender influences food waste. Regarding education, Secondi et al. [50] are amongst the first to indicate causality between level of education and the amount of food wasted. The lower the level of education, the smaller the amount of food waste generated. Focusing on socio-economic status and standards of living various studies state that higher income households waste more than poorer households [46,50]. This study will assess whether and to what extent the previously mentioned causalities can(not) be confirmed:

**Hypothesis** **8a** **(H8a).** *Household income has a positive impact on FW.*

**Hypothesis** **8b** **(H8b).** *Educational level has a negative influence on FW.*

**Hypothesis** **8c** **(H8c).** *Household composition has a positive impact on FW.*

**Hypothesis** **8d** **(H8d).** *Children’s age in a household has a positive influence on FW.*

**Hypothesis** **8e** **(H8e).** *Age has a negative impact on FW.*

**Hypothesis** **8f** **(H8f).** *Gender influences FW.*

## 3. Materials and Methods

### 3.1. Participants

The study focused on Dutch consumers (partly) responsible for the household’s food purchasing. Participants were recruited via online and mobile platforms, i.e., Facebook, LinkedIn, and Whatsapp. The survey link was sent to potential participants who were asked to forward the link to family and friends (snowball sampling). To attain the target group a filter question was included: only those participants answering ‘yes’ to the opening question of being (partly) responsible for either the purchasing or cooking of food in their household were withdrawn and able to continue the study.

### 3.2. Procedure

Data was collected during August and September 2018 via an online questionnaire. The questionnaire was set up in English and distributed through digital platforms (Facebook, LinkedIn, WhatsApp). The questionnaire was pre-tested with five persons to check wording, clarity, and interpretation of all questions. Based on feedback some questions were re-formulated or slightly modified (see Appendix A for the final questionnaire).

- Food management behaviors. To measure household food management behaviors associated with food waste, this study used validated measurements [16,17] and added relevant items based on Romani et al. [14]. Food planning, purchase, storage, preparation, and leftover consumption in relation to food waste were presented (e.g., ‘How often do you make a list of the food you want to buy prior to your shopping trip?’), with answers to be indicated on a 5-point Likert scale ranging from ‘never’ (1), over ‘sometimes’ (3) to ‘always’ (5).
- Intention not to waste food. This concept was measured using three items [16] following the theory of planned behavior guidelines [31]. The items were to be rated on a 7-point Likert scale (from ‘strongly disagree’ (1) to ‘strongly agree’ (7)).
- (Lack of) Concern about food waste. A scale of general attitude toward food waste was used consisting of three items to be rated on a 7-point Likert scale (from ‘strongly disagree’ (1) to ‘strongly agree’ (7)) [17]. The items refer to throwing away food in relation to environmental concern.
- Reported amount of food waste. Self-reported food waste behavior was measured using a 5-point Likert scale [17] ranging from ‘not at all’ (1), ‘less than a tenth’ (2), ‘more than a tenth but less than a quarter’ (3), ‘more than a quarter but less than half’ (4) to ‘more than half’ (5). The items refer to food waste in general and to four specific subcategories, i.e., dairy, fresh fruit and vegetables, meat and fish, and bakery products.
- Socio-demographics. According to Secondi et al. [50] socio-demographic characteristics influence food waste behavior, therefore the survey held questions on age, gender, household income, household composition, and educational level.

**Figure 1:**
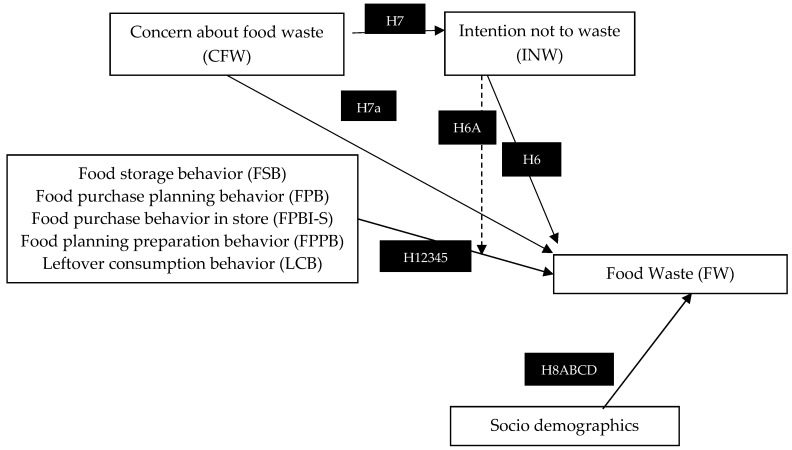
Conceptual model of Dutch households’ reported food waste.

Figure 1 represents the conceptual model including the constructs and hypotheses.

Both measurements and structural models developed in this study were analyzed via structural equation modeling (SEM). In behavioral sciences data often are not normally distributed, can be limited, need more complex models [51], or have models that have less theoretical backing [52]. Whereas covariance-based structural equation modeling (CB-SEM) treats data as multiple linear regressions, partial least square modeling (PLS-SEM) is variance-based and realizes correlations between constructs and their items (measuring models) and linear regressions between constructs (structural model). As, in the current study, the goal is to explain the reported amount of food waste through the constructs of food management behaviors and food waste awareness PLS-SEM is most suitable [53]. Table 1 shows the steps to be taken when evaluating the two submodels in SEM, i.e., the measurement models and the structural model [54].

When evaluating PLS-SEM results, first the measurement models need to be examined. If they meet all required criteria the next step is to assess the structural model [53,54]. The criteria to be met differ for formative and reflective models. In a formative model, the items cause the construct and thus a change in one item does not necessarily imply a change in others. In this study, the model is reflective, meaning that all items depend on the construct and are highly correlated to one another [51].

For the analyses of PLS-SEM and thus evaluation of the measurement models and structural model SmartPLS software was used [54].

## 4. Results

### 4.1. Participants

A total of 211 Dutch participants (partly) responsible for the household shopping and/or cooking completed the questionnaire, with ages ranging from 20 to 66 years old (M = 36.69, SD = 12.17). Twenty-eight percent of the participants were men. The majority of the participants held a bachelor’s degree (38%), followed by an intermediate vocational training degree (26%), a master’s degree (23%), an associate degree (6%), an elementary school degree (4%), and other degrees (3%).

The household composition of the participants was defined as follows: a total of 25 single person households, 100 households with no (more) children living in, and 86 households with children. Thirty-five percent indicated having a household net income of more than €5000, followed by 25% having in between €2000 and €3000, 19% disposed of a household net income of €3000–€4000, 11% stated to have €2000 or less, and 10% preferred not to answer the question.

Gender, Household Income, Household Composition, Education, and Age of Children had no significant role in influencing Food Waste and therefore were left out in further analyses. Age, however, appeared to correlate negatively (*r* = −0.17, *p* < 0.001) with Food Waste and thus will be included in analyses of both measurement and structural models.

### 4.2. The Measurement Models

The first step to assess reflective measurement models is to evaluate the item loadings. According to Risher et al. [55] loadings above 0.71 are recommended indicating that the construct explains more than 50% of the item’s variance and thus gives sufficient item reliability. All item loadings are well above the threshold value of 0.70, supporting reliability of the construct measures (Table 2). The second step is testing the internal consistency reliability which includes evaluation of the composite reliability (CR) [53,54]. CR values vary between 0 and 1 and, in general, higher CR values lead to higher reliability levels. Values as of 0.70 can be seen as satisfactory, but CR values cannot exceed 0.95 because then the items would be measuring the same phenomenon [53]. In this study, items with CR values well below the 0.70 value threshold were discarded ensuring an acceptable internal consistency reliability of the construct measures. None of the socio-demographic items met the above-mentioned criteria except Age (hence the CR value of 1, meaning that Age represents the socio-demographics).

The third step of the reflective measurement model assessment addresses validity and is studied through both convergent and discrimant validity. “Convergent validity is the extent to which a construct converges to explain the variance of its items” [55] (p. 9). The measure used for convergent validity here is Average Variance Extracted (AVE) and should be greater than or equal to 0.50 [53]. All AVE values exceed this value and thus convergent validity of each construct measure is established.

To assess discriminant validity (i.e., in how far is a construct substantially distinct from the other constructs) in a reflective measurement model all item loadings and cross-loadings need to be examined. The item loadings should be higher for the latent variable (i.e., construct measure) they are part of than for any other construct [53]. Table 3 shows that all loadings exceed the cross-loadings indicating that discriminant validity is established.

In addition, the Fornell–Larcker Criterion was taken into account to corroborate the discriminant validity outcome. This criterion assumes that each construct shares more variance with its own items than with any other construct [53]. The diagonal represents AVE square roots while the off-diagonal shows the correlations between the constructs. “To meet the Fornell–Larcker Criterion the square root of every constructs’ AVE should be higher than the constructs’ highest correlation with any other construct” [53] (p. 126) (Table 4, in bold).

An alternative approach to measuring discriminant validity is looking at the Heterotrait-Monotrait ratio (HTMT) of the item correlations [56]. In order to establish adequate discriminant validity, the HTMT value may not surpass 0.90, correlations with a value close to 1 indicate a lack of discriminant validity. Table 5 confirms that all HTMT values are below this threshold.

When evaluating the measurement models, we can confirm that the construct measures are reliable and valid.

### 4.3. The Structural Model

The process of assessing the structural model starts with examining collinearity issues (i.e., highly correlated independent variables). Scores of the predictor constructs in a partial regression are needed to calculate the Variance Inflation Factor (VIF). All VIF values are well below the threshold of value 5 (i.e., all VIF < 1.5) which suggests no issues with collinearity [53]. That is, the constructs in our model do not overlap and can be considered as reliable (Table 6).

Next, the R^2^ value of the endogenous construct(s)—constructs explained by the relationships in the model—is to be examined. The R^2^ value measures the variance, explained in each of the endogenous constructs, and thus the predictive accuracy of the structural model [53]. More specifically, this coefficient of determination indicates the variation in the reported amount of food waste, explained by the independent variables. A higher R^2^ value means more variability is explained by the structural model. The effect ranges from 0 to 1, with a value of 1 indicating full predictive accuracy. In this study, R^2^ has a value of 0.38 which—in studies related to consumer behavior—can be described as moderate to substantial explanatory power of the model.

In addition to the R^2^ values effect size (f^2^) can be measured. f^2^ is determined by the change in R^2^ when a construct is eliminated from the structural model [53]. The effect sizes (f^2^) are displayed in Table 7. Guidelines indicate that the current results show no effects (i.e., f^2^ values < 0.02) [57] for CFW, FPPB, FPB, FSB, INW × FPB, INW × FPBI-S, INW × FSB, INW × LCB, and LCB. The f^2^ values for FPBI-S, INW × FPPB, socio-demographics (i.e., Age) and INW indicate a small (f^2^ ≤ 0.02) to moderate (f^2^ ≤ 0.15) effect [53].

Path coefficients are examined indicating the strength of the relationship between constructs (Table 8). A value close to 1 suggests a strong positive relationship whereas a value closer to 0 suggests a weak relationship [53]. The results presented below indicate a weak although positive significant relation between FW and FPBI-S (β = 0.31, *p* < 0.001). That is, when participants do not buy more food products than needed or when they do not buy food products they did not plan on buying (i.e., control their buying behavior) the lower the reported amount of food waste. Negative, quite weak but significant relations were found between FW and INW (β = −0.34, *p* < 0.001), FW and Age (β = −0.18, *p* < 0.001), and CFW and INW (β = −0.33, *p* < 0.001). The higher the intent not to waste food, the lower the reported amount of food wasted. Additionally, the younger the participants the higher the reported amount of food waste. Similarly, the higher the lack of concern about food waste, the smaller the participant’s intention to reduce food waste. In addition, a positive significant moderating effect is established of FPPB on FW via INW (β = 0.17, *p* = 0.02). Thus, the interaction term has a positive effect on FW. Meaning, the higher the intention not to waste food, the stronger the relationship between FPPB and FW. The greater the participant’s intent to reduce their food waste the more likely they plan their weekly menu leading to a smaller amount of food waste.

**Figure 2:**
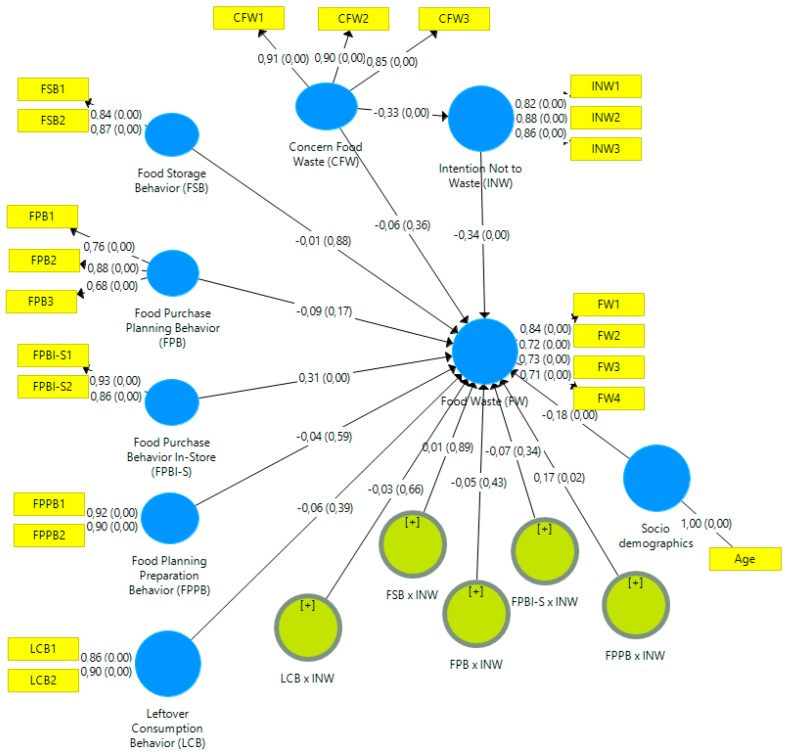
Structural model with path coefficients and levels of significance.

Figure 2 presents the final model.

### 4.4. Hypotheses Tests

Table 9 gives an overview of the tested and confirmed hypotheses.

## 5. Discussion

Food production and consumption—and food waste in particular—are responsible for striking negative effects on the environment [1]. Food losses and food waste occur throughout the entire food system, however, households are responsible for the largest amount of food waste. Therefore, this study aimed to identify how consumer behavior in daily food provisioning affects food waste, and what food management behaviors may be tackled in reducing food waste.

Validated scales from previous research were pre tested and adapted to fit this study. The results provided insights proven different than predicted. Analyses resulted in the acceptance of five hypotheses. Food Purchase Behavior In-Store (FPBI-S) has a significant, positive effect on food waste (FW). That is, the more consumers rely on their shopping behavior (e.g., buying too much in the store, having certain shopping routines) the more they will end up wasting food. FPBI-S appears to be the only food waste management behavior that has an impact on reported food waste, which is in line with [16,17], identifying that a substantial variance of food waste is explained by shopping routines.

According to TPB, household food waste behavior is negatively related to the intention to reduce household food waste (INW) [40,58]: the higher the intent to reduce food waste (or the intent not to waste food) the lower the amount of actual food waste was reported. Yet, in [17] no relationship between INW and FW was found.

Findings in the current research, however, indicate the opposite: INW has a significant, negative impact on FW. The higher the consumer’s intent not to waste, the lower their reported food waste. This result confirms previous work [16] identifying a significant, although weak, negative effect of INW on FW.

In addition to earlier research on food waste behaviors this study examined the moderating impact of INW on different food waste management behaviors. A significant, positive relationship is found between FPPB and (reported amount of) FW, moderated by the intent consumers have not to waste food. More specifically, the greater the consumer’s intent not to waste food, the greater the relationship between FPPB and FW. When consumers plan their (food) purchases they will waste less food, positively influenced by their intent not to waste. This research confirms that the lack of planning for food preparation appears to be one of the most significant barriers to reducing food waste to a minimum. In general, the low scores on planning are an indication of the general inability felt by consumers to plan their meals in advance and to organize a weekly menu. In [16] the planning routines made only an indirect contribution to food waste, unlike the findings from [17].

In line with [17] this research also identified a significant, negative impact of Concern about food waste (CFW) on INW. The TPB context [31] states that people share an ideal not to waste food, thus measuring directly whether people think that wasting food is good or bad. Consumer’s (lack of) concern towards food waste determine their intention not to waste food, as based on the TPB model [17]. However, other research [16] states that consumers did not make any connection between food waste and environmental concerns. The current study confirms a (weak) relationship between CFW and INW.

Age is the only socio-demographic variable having a significant, negative influence on the reported amount of FW. The older the consumers the less they reported to be wasting. This result corroborates that reported in [16,17]; age correlated negatively with reported food waste although the correlation found was relatively small.

### 5.1. Managerial Implications

Overall, the results provide important insights for (retail) managers and policymakers interested in designing initiatives aiming at reducing food waste at household level. The finding that food purchase behavior in-store impacts (reported amount of) food waste in consumers is of great importance for (grocery) retailers. Retailers could help reduce food waste by selling in smaller quantities, so consumers are not obliged to buy more than needed. Marketing communication in-store (e.g., information screens) could help raise the consumer’s awareness regarding food waste and hence remind them to buy in a durable manner. In addition, an important role in sustainable shopping and eating behavior is reserved for (food) marketers: experimenting with offered package size or offered promotions are marketing actions that can help tackle food waste.

### 5.2. Limitations

First, this study relies on the results of self-reported behavior. That is, participants were asked to write down their estimated food waste (food thrown away without consumption) in a regular week. Due to the negative connotation of wasting food, participants may have possibly answered in a socially desirable manner. Additionally, consumers may not be accurately aware of the quantity of wasted food and hence underestimate the amount of food they throw away per week. Giordano et al. [59] compared different methods to gather information about food waste. They found out that reported food waste quantities are heavily biased, as they are significantly higher in the diary method, compared to the questionnaires approach that only reports one-third of food waste determinants. The low food waste quantities reported through questionnaires was also evinced by Fanelli [60], in a survey among 1058 Italian consumers, who concluded that perceived food waste quantities as mentioned by the respondents were low.

Second, data were collected through an online survey which was distributed via social media networks. Participants thus were consumers within the same network, biasing the variance in our socio-demographic data. In addition, only those consumers with a certain interest in the survey’s topic or willingness to participate engaged in the study, creating a self-selection bias. This may also explain the divergence of results from other studies. The data can therefore only be interpreted from the perspective of a convenience sample. Consequently, representativeness was never this study’s goal. In order to generalize the results a study with more demographical variety is needed.

### 5.3. Further Research

Considering that consumers purchase and make decisions on what is available to them, food retailers could develop actions to help them reduce food waste, e.g., by developing better food packaging. Previous studies already show the great potential of packaging in preventing and reducing food waste. In the light of the current results it would be interesting to explore how retailers and food marketers see their role.

From another angle, the influence of online grocery shopping on food waste should be studied. In 2017, the share of Dutch consumers buying online food was the highest in the European Union, with 29% [61]. Comparing the effect of online versus in-store grocery shopping on waste behavior would therefore be a fascinating subsequent study.

## Figures and Tables

**Table 1:** Stepwise process to evaluate model results.

Step 1. Evaluation of Reflective Measurement Models
a) Internal consistency reliability:
item loadings
composite reliability
b) Examining validity
convergent validity (metric used is the Average Variance Explained)
discriminant validity (metric used is the Fornell–Larcker criterium or—the more precise—heterotrait-monotrait (HTMT) ratio of the correlations)
Step 2. Evaluation of the structural model
a) Coefficients of determination (R^2^) b) Predictive relevance (Q^2^) c) Size and significance of path coefficients d) f^2^ effect sizes e) q^2^ effect sizes

**Table 2:** Construct measures, items, loadings, reliability, and validity.

Construct Measure	Item	Loading	Composite Reliability (CR)	Average Variance Extracted (AVE)
Concern Food Waste (CFW)			0.917	0.787
	CFW1	0.907 ***		
	CFW2	0.895 ***		
	CFW3	0.857 ***		
Food Planning Preparation Behavior (FPPB)			0.907	0.83
	FPPB1	0.922 ***		
	FPPB2	0.9 ***		
Food Purchase Behavior In-Store (FPBI-S)			0.888	0.799
	FPBI-S1	0.865 ***		
	FPBI-S2	0.796 ***		
	FPBI-S3	0.516		
	FPBI-S4	0.629		
Food Purchase Planning Behavior (FPB)			0.82	0.605
	FPB1	0.739 ***		
	FPB2	0.876 ***		
	FPB3	0.667 ***		
	FPB4	0.366		
Food Storage Behavior (FSB)			0.843	0.729
	FSB1	0.72 ***		
	FSB2	0.748 ***		
	FSB3	0.606		
	FSB4	0.512		
Food Waste (FW)			0.835	0.56
	FW1	0.834 ***		
	FW2	0.722 ***		
	FW3	0.73 ***		
	FW4	0.703 ***		
Intention Not to Waste (INW)			0.889	0.727
	INW1	0.817 ***		
	INW2	0.876 ***		
	INW3	0.864 ***		
Leftover Consumption Behavior (LCB)			0.873	0.775
	LCB1	0.858 ***		
	LCB2	0.902 ***		
Age	Age	1	1	1

Note: *** indicate significance at *p* < 0.01.

**Table 3:** Loadings and cross-loadings of all construct measures and their items.

	CFW	FPB	FPBI−S	FPPB	FSB	INW	LCB	FW	Socio-Demographics
CFW1	0.907	0.061	0.042	0.025	0.049	−0.277	−0.15	0.104	−0.151
CFW2	0.895	0.057	0.073	0.037	0.014	−0.298	−0.092	0.124	−0.163
CFW3	0.857	−0.061	−0.034	0.033	0.007	−0.31	−0.074	0.073	−0.07
FPB1	0.065	0.761	−0.158	0.435	0.129	0.146	0.128	−0.178	0.048
FPB2	0.011	0.879	−0.279	0.367	0.246	0.193	0.115	−0.247	0.049
FPB3	−0.03	0.68	−0.099	0.185	0.202	0.155	0.247	−0.176	−0.099
FPBI−S1	0.043	−0.206	0.926	−0.177	−0.126	−0.145	−0.08	0.439	−0.139
FPBI−S2	0.004	−0.234	0.861	−0.181	−0.141	−0.05	−0.039	0.326	−0.16
FPPB1	0.051	0.417	−0.157	0.922	0.23	0.042	0.084	−0.153	0.08
FPPB2	0.012	0.354	−0.209	0.9	0.137	0.048	0.069	−0.136	−0.037
FSB1	0.052	0.256	−0.155	0.235	0.841	0.108	0.114	−0.1	0.021
FSB2	−0.006	0.179	−0.099	0.119	0.867	0.157	0.163	−0.108	−0.138
INW1	−0.192	0.213	−0.136	0.052	0.177	0.817	0.377	−0.352	0.022
INW2	−0.322	0.148	−0.073	0.004	0.118	0.875	0.27	−0.308	0.117
INW3	−0.329	0.189	−0.093	0.07	0.11	0.864	0.323	−0.315	0.069
LCB1	−0.112	0.113	−0.044	0.052	0.152	0.336	0.858	−0.174	−0.027
LCB2	−0.097	0.231	−0.076	0.093	0.138	0.328	0.902	−0.207	0.072
FW1	0.068	−0.216	0.433	−0.163	−0.113	−0.317	−0.257	0.837	−0.274
FW2	0.101	−0.234	0.283	−0.127	−0.019	−0.324	−0.099	0.717	−0.148
FW3	0.073	−0.107	0.19	−0.071	−0.038	−0.247	−0.164	0.729	−0.207
FW4	0.104	−0.212	0.345	−0.095	−0.179	−0.242	−0.104	0.704	−0.185
Age	−0.143	0.006	−0.165	0.027	−0.073	0.084	0.03	−0.277	1

**Table 4:** Fornell–Larcker Criterion (in bold).

	CFW	FPPB	FPBI-S	FPB	FSB	FW	INW	LCB	Age
Concern Food Waste (CFW)	0.887								
Food Planning Preparation Behavior (FPPB)	0.036	0.911							
Food Purchase Behavior In-Store (FPBI-S)	0.029	−0.199	0.894						
Food Purchase Planning Behavior (FPB)	0.019	0.425	−0.242	0.778					
Food Storage Behavior (FSB)	0.026	0.205	−0.147	0.253	0.854				
Food Waste (FW)	0.113	−0.159	0.435	−0.262	−0.122	0.749			
Intention Not to Waste (INW)	−0.334	0.049	−0.116	0.214	0.156	−0.38	0.853		
Leftover Consumption Behavior (LCB)	−0.117	0.085	−0.07	0.2	0.164	−0.218	0.376	0.88	
Age	−0.143	0.027	−0.165	0.006	−0.073	−0.277	0.084	0.03	1

Note: AVE in bold.

**Table 5:** Heterotrait-Monotrait ratio.

	CFW	FPPB	FPBI-S	FPB	FSB	FW	INW × FPB	INW × FPBI-S	INW × FPPB	INW × FSB	INW × LCB	INW	LCB	Age
CFW														
FPPB	0.045													
FPBI-S	0.066	0.26												
FPB	0.092	0.578	0.327											
FSB	0.048	0.289	0.218	0.385										
FW	0.145	0.198	0.548	0.359	0.195									
INW × FPB	0.016	0.034	0.05	0.216	0.075	0.066								
INW × FPBI-S	0.024	0.019	0.237	0.046	0.208	0.195	0.039							
INW × FPPB	0.158	0.063	0.064	0.073	0.035	0.183	0.369	0.29						
INW × FSB	0.029	0.025	0.205	0.087	0.098	0.067	0.403	0.331	0.197					
INW × LCB	0.077	0.014	0.109	0.057	0.169	0.07	0.456	0.246	0.103	0.448				
INW	0.392	0.063	0.141	0.29	0.22	0.488	0.211	0.153	0.04	0.168	0.44			
LCB	0.153	0.109	0.09	0.298	0.244	0.286	0.066	0.114	0.019	0.179	0.164	0.499		
Age	0.155	0.072	0.192	0.103	0.117	0.315	0.017	0.125	0.089	0.121	0.086	0.09	0.067	

**Table 6:** Collinearity statistics (Variance Inflation Factor (VIF))—inner VIF values.

	CFW	FPPB	FPBI-S	FPB	FSB	FW	INW × FPB	INW × FPBI-S	INW × FPPB	INW × FSB	INW × LCB	INW	LCB	Age
CFW						1.209								
FPPB						1.273								
FPBI-S						1.207								
FPB						1.417								
FSB						1.175								
FW														
INW × FPB						1.588								
INW × FPBI-S						1.434								
INW × FPPB						1.45								
INW × FSB						1.564								
INW × LCB						1.679								
INW						1.64								
LCB						1.217								
Age						1.088								

**Table 7:** Effect sizes (f^2^).

	CFW	FPPB	FPBI-S	FPB	FSB	INW × FPB	INW × FPBI-S	INW × FPPB	INW × FPB	INW × LCB	INW	LCB	Age
FW	0.005	0.002	0.13	0.009	0	0.003	0.007	0.027	0	0.001	0.117	0.004	0.047
INW	0.125												

**Table 8:** Path coefficients (β) and significances (*p*).

	Path Coefficients (β)	*p* Values
CFW → FW	−0.059	0.359
CFW → INW	−0.333	0.000
FPPB → FW	−0.037	0.591
FPBI-S → FW	0.311	0.000
FPB → FW	−0.089	0.168
FSB → FW	−0.01	0.883
INWxFPB → FW	−0.051	0.430
INWxFPBI-S → FW	−0.066	0.343
INWxFPPB → FW	0.173	0.017
INWxFSB → FW	0.011	0.888
INWxLCB → FW	−0.03	0.660
INW → FW	−0.343	0.000
LCB → FW	−0.058	0.389
Age → FW	−0.177	0.001

Note: significant *p*-values are presented in bold.

**Table 9:** Confirmed hypotheses.

Hypotheses	Variables	Confirmed	Not-Confirmed
H1	Food storage behavior (FSB)		x
H2	Food purchase planning behavior (FPB)		x
H3	Food purchase behavior in-store (FPBI-S)	x	
H4	Food preparation planning behavior (FPPB)		x
H5	Leftover consumption behavior (LCB)		x
H6	Intention Not to Waste (INW)	x	
H6a	Intention Not to Waste as moderator	x *	
H7	Concern about food waste (CFW) → INW	x	
H7a	Concern about food waste (CFW) → Food Waste (FW)		x
H8a	Household income		x
H8b	Educational level		x
H8c	Household composition		x
H8d	Children’s age in household		x
H8e	Age	x	
H8f	Gender		x

Note: * Partly confirmed.

## Appendix A

Questionnaire

**Table A1:** Food management behaviors.

Item	Statements + Label	Source
**Please answer the follow questions, ranging from 1 (never) to 5 (always).**		Stefan et al. 2013; Stancu et al. 2016; Romani et al. 2018.
	**Food Storage Behaviors—FSB** FSB1_How often do you check your fridge? FSB2_How often do you check your pantry? FSB3_How often do you use food with limited expiry dates compared to food with extended expiry dates? * FSB4_How often do you check the expiry date of food in the pantry? *	
	**Food Purchasing Behaviors—FPB** FPB1_How often do you make a list of the food you want to buy prior to your shopping trip? FPB2_How often do you check your food inventories prior to your shopping trip? FPB3_How often do you avoid buying things that you already have in the pantry? FPB4_How often do you check the expiry date of the products when shopping? *	
	**Food Purchasing Behavior in store—FPBI-S** FPBIS1_How often do you buy too many food products (more than you need) when you go shopping? * FPBIS2_How often do you buy food items that you did not plan to buy? * FPBIS3_How often do you buy larger amounts of food because shops are offering bargains? * FPBIS4_How often do you buy food in packages that are too big for your household’s needs? *	
	**Food Planning Preparation Behaviors—FPPB** FPPB1_How often do you plan your meals several days in advance? FPPB2_How often do you follow a weekly menu?	
	**Leftover Consumption Behaviors—LCB** LCB1_How often do you eat leftovers cold, or just reheated? LCB2_How often do you store the leftovers in appropriate conditions so that they will last and be used adequately? * *Reverse coding so that all scales correspond to the same direction of wording.	

**Table A2:** Intention not to waste food.

Item	Statements + Label	Source
**Please answer the following questions thinking about the near future (e.g., next one/two weeks) and your household. Scale: strongly disagree (1) to strongly agree (7).**		Stefan et al. 2013.
	INW1_I intend not to throw food away INW2_My goal is not to throw food away INW3_I will try not to throw food away	

**Table A3:** Concern about food waste.

Item	Statements + Label	Source
**Please answer the following questions thinking about the near future (e.g., next one/two weeks) and your household. Scale: strongly disagree (1) to strongly agree (7).**		Stefan et al. 2013.
	CFW1_ I do not really worry about the environmental impact of the food that I throw away CFW2_I do not really worry about the impact of my food waste on the distribution of resources in the world CFW3_I do not really worry about the amount of food that I throw away.	

**Table A4:** Reported amount of food waste.

Item	Statements + Label	Source
**For each of the items shown, please select an answer that indicates how much of what you buy gets thrown away, in a regular week? “not at all” (1), “less than a tenth” (2), “more than a tenth but less than a quarter” (3), “more than a quarter, less than a half” (4), and “more than a half” (5)**		Stefan et al. 2013.
	How much … would you say that you throw away what you buy and/or grow, in a regular week? FW1_Fresh fruit and vegetables FW2_Dairy product FW3_Meat and fish FW4_Bread and other bakery products	

**Table A5:** Socio-demographics.

Age_ Gender _Male _Female _Other _Prefer not to say	**Secondi et al. 2015.**
University degree _ Elementary and Secondary school _ Middle-level applied education (MBO) _Associate degree _Bachelor’s degree _ Masters’ degree _ Doctorate degree _Other	
Household Composition _Single-person household _Household without kids (or kids who do not live at home anymore) _Household with one kid _Household with two kids _Household with three kids _Household with four kids or more	
Average age of kids within household _Under 5 years old _Between 5 and 10 years old _Between 10 and 15 years old _Older than 15, younger than 20 _Older than 20 _No kids or no kids who live at home	
Net income household (monthly) _<€1000 _ €1001/€2000 _ €2001/€3000 _ €3001/€4000 _>€4000 _ Prefer not to say	

## References

[1] Holt A.R., Alix A., Thompson A., Maltby L. (2016). Food production, ecosystem services and biodiversity: We can’t have it all everywhere. Sci. Total Environ..

[2] Stenmarck A., Jensen C., Quested T., Moates G. Estimates of European Food Waste Levels. http://eufusions.org/phocadownload/Publications/Estimates%20of%20European%20food%20waste%20levels.pdf.

[3] Block L.G., Keller P.A., Vallen B., Williamson S., Birau M.M., Grinstein A., Haws K.L., LaBarge M.C., Lamberton C., Moore E.S. (2016). The squander sequence: Understanding food waste at each stage of the consumer decision-making process. J. Public Policy Mark..

[4] Searchinger T., Hanson C., Ranganathan J., Lipinski B., Waite R., Winterbottom R., Dinshaw A., Heimlich R. (2013). Creating a Sustainable Food Future: Interim Findings. A Menu of Solutions to Sustainably Feed More than 9 Billion People by 2050.

[5] Eurostats (2011). Food: From Farm to Fork Statistics.

[6] Jörissen J., Priefer C., Bräutigam K.-R. (2015). Food waste generation at household level: Results of a survey among employees of two European research centers in Italy and Germany. Sustainability.

[7] Kosseva M.R., Webb C. (2013). Food Industry Wastes—Assessment and Recuperation of Commodities.

[8] Ponis S.T., Papanikolau P., Katimertzoglou P., Ntalla A., Xenos K.I. (2017). Household food waste in Greece: A questionnaire survey. J. Clean. Prod..

[9] Parfitt J., Barthel M., Macnaughton S. (2010). Food waste within food supply chains: Quantification and potential for change to 2050. Philos. Trans. R. Soc. B.

[10] Scherhaufer S., Moates G., Hartikainen H., Waldron K., Obersteiner G. (2018). Environmental impacts of food waste in Europe. Waste Manag..

[11] Schanes K., Dobernig K., Gözet B. (2018). Food waste matters—A systematic review of household food waste practices and their policy implications. J. Clean. Prod..

[12] Eurobarometer Food Waste and Date Marking. https://data.europa.eu/euodp/en/data/dataset/S2095_425_ENG.

[13] Porpino G. (2016). Household food waste behavior: Avenues for future research. J. Assoc. Consum. Res..

[14] Romani S., Grappi S., Bagozzi R.P., Barone A.M. (2018). Domestic food practices: A study of food management behaviors and the role of food preparation planning in reducing waste. Appetite.

[15] Stöck S., Niklaus E., Dorn M. (2018). Call for testing interventions to prevent consumer food waste. Resour. Conserv. Recycl..

[16] Stancu V., Haugaard P., Lähteenmäki L. (2016). Determinants of consumer food waste behavior: Two routes to food waste. Appetite.

[17] Stefan V., van Herpen E., Tudoran A.A., Lähteenmäki L. (2013). Avoiding food waste by Romanian consumers: The importance of planning and shopping routines. Food Qual. Prefer..

[18] FAO Food Wastage Footprint: Impacts on Natural Resources: Summary Report. http://www.fao.org/docrep/018/i3347e/i3347e.pdf.

[19] Mourad M. (2016). Recycling, recovering and preventing “food waste”: Competing solutions for food systems sustainability in the United States and France. J. Clean. Prod..

[20] Niles M., Ahuja R., Barker T., Esquivel J., Gutterman S., Heller M., Vermeulen S. (2018). Climate change mitigation beyond agriculture: A review of food system opportunities and implications. Renew. Agric. Food Syst..

[21] Thyberg K.L., Tonjes D.J. (2016). Drivers of food waste and their implications for sustainable policy development. Resour. Conserv. Recycl..

[22] Schanes K., Giljum S., Hertwich E. (2016). Low carbon lifestyles: A framework to structure consumption strategies and options to reduce carbon footprints. J. Clean. Prod..

[23] Blanke M. (2015). Challenges of reducing fresh produce waste in Europe—From farm to fork. Agriculture.

[24] Wilson N.L.W., Rickard B.J., Saputo R., Ho S.-T. (2017). Food waste: The role of date labels, package size, and product category. Food Qual. Prefer..

[25] Roodhuyzen D.M.A., Luning P.A., Fogliano V., Steenbekkers L.P.A. (2017). Putting together the puzzle of consumer food waste: Towards an integral perspective. Trends Food Sci. Technol..

[26] Hanssen O.J., Syversen F., Stø E. (2016). Edible food waste from Norwegian households—Detailed food waste composition analysis among households in two different regions in Norway. Resour. Conserv. Recycl..

[27] Quested T.E., Marsh E., Stunell D., Parry A.D. (2013). (WRAP). Spaghetti soup: The complex world of food waste behaviors. Resour. Conserv. Recycl..

[28] Bravi L., Murmura F., Savelli E., Viganò E. (2018). Motivations and actions to prevent food waste among young Italian consumers. Sustainability.

[29] Calvo-Porral C., Medín A.F., Losada-López C. (2016). Can Marketing help in tackling food waste? Proposals in Developed Countries. J. Food Prod. Mark..

[30] Janssen A.M., Nijenhuis-de Vries M.A., Boer E.P., Kremer S. (2017). Fresh, frozen, or ambient food equivalents and their impact on food waste generation in Dutch households. Waste Manag..

[31] Ajzen I. (1991). The theory of planned behavior. Organ. Behav. Hum. Decis. Process..

[32] Aschemann-Witzel J., de Hooge I., Amani P., Bech-Larsen T., Oostindjer M. (2015). Consumer-related food waste: Causes and potential for action. Sustainability.

[33] Bolton L.E., Alba J.W. (2012). When less is more: Consumer aversion to unused utility. J. Consum. Psychol..

[34] Graham-Rowe E., Jessop D.C., Sparks P. (2014). Identifying motivations and barriers to minimising household food waste. Resour. Conserv. Recycl..

[35] Quested T.E., Parry A.D., Easteal S., Swannell R. (2011). Food and drink waste frome households in the UK. Nutr. Bull..

[36] Porpino G., Parente J., Wansink B. (2015). Food waste paradox: Antecedents of food disposal in low income households. Int. J. Consum. Stud..

[37] Evans D. (2011). Blaming the consumer—Once again: The social and material contexts of everyday food waste practices in some English households. Crit. Public Health.

[38] Verplanken B., Aarts H., van Knippenberg A., Moonen A. (1998). Habit versus planned behavior: A field experiment. Br. J. Soc. Psychol..

[39] Setti M., Banchelli F., Falasconi L., Segrè A., Vittuari M. (2018). Consumers’ food cycle and household waste. When behaviors matter. J. Clean. Prod..

[40] Graham-Rowe E., Jessop D.C., Sparks P. (2015). Predicting household food waste reduction using an extended theory of planned behavior. Resour. Conserv. Recycl..

[41] Sheeran P., Stroebe W., Hewstone M. (2002). Intention behavior relations: A conceptual and empirical review. European Review of Social Psychology.

[42] Grunert K.G., Hieke S., Wills J. (2014). Sustainability labels on food products: Consumer motivation, understanding and use. Food Policy.

[43] Le Borgne G., Sirieix L., Costa S. (2018). Perceived probability of food waste: Influence on consumer attitudes towards and choice of sales promotions. J. Retail. Consum. Serv..

[44] Visschers V.H.M., Wickli N., Siegrist M. (2016). Sorting out food waste behavior: A survey on the motivators and barriers of self-reported amounts of food waste in households. J. Environ. Psychol..

[45] Diaz-Ruiz R., Costa-Font M., Gil J.M. (2018). Moving ahead from food-related behaviors: An alternative approach to understand household food waste generation. J. Clean. Prod..

[46] Principato L., Secondi L., Pratesi C.A. (2015). Reducing food waste: An investigation on the behavior of Italian youths. Br. Food J..

[47] Koivupuro H.K., Hartikainen H., Silvennoinen K., Katajajuuri J.M., Heikintalo N., Reinikainen A., Jalkanen L. (2012). Influence of socio-demographical, behavioural and attitudinal factors on the amount of avoidable food waste generated in Finnish households. Int. J. Consum. Stud..

[48] Parizeau K., von Massow M., Martin R. (2015). Household-level dynamics of food waste production and related beliefs, attitudes, and behaviors in Guelph, Ontario. Waste Manag..

[49] OECD Family Size and Household Composition. https://www.oecd.org/els/family/SF_1_1_Family_size_and_composition.pdf.

[50] Secondi L., Principato L., Laureti T. (2015). Household food waste behavior in EU-27 countries: A multilevel analysis. Food Policy.

[51] Haenlein M., Kaplan A.M. (2004). A beginners’ guide to partial least squares analysis. Underst. Stat..

[52] Ringle C.M., da Silva D., Bido D.S. (2014). Structural equation modeling with the SmartPLS. Braz. J. Mark..

[53] Hair J.F., Hult G.T.M., Ringle C.M., Sarstedt M. (2017). A Primer on Partial Least Squares Structural Modelling (PLS-SEM).

[54] Wong K.K. (2019). Mastering Partial Least Squares Structural Equation Modeling (Pls-sem) with Smartpls in 38 Hours.

[55] Hair J.F., Risher J.J., Sarstedt M., Ringle C.M. (2019). When to use and how to report the results of PLS-SEM. Eur. Bus. Rev..

[56] Henseler J., Ringle C.M., Sarstedt M. (2015). A new criterion for assessing discriminant validity in variance-based structural equation modeling. J. Acad. Mark. Sci..

[57] Cohen J. (1988). Statistical Power Analysis for the Behavioral Sciences.

[58] Russell S.V., Young C.W., Unsworth K.L., Robinson C. (2017). Bringing habits and emotions into food waste behavior. Resour. Conserv. Recycl..

[59] Giordano C., Alboni F., Falasconi L. (2019). Quantities, determinants, and awareness of households’ food waste in Italy: A comparison between diary and questionnaires quantities. Sustainability.

[60] Fanelli R.M. (2019). Using causal maps to analyse the major root causes of household food waste: Results of a survey among people from Central and Southern Italy. Sustainability.

[61] EU Sustainable Development in the European Union. https://ec.europa.eu/eurostat/documents/3217494/8461633/KS-04-17-780-EN-N.pdf.

